# Rugby Fans in Training New Zealand (RUFIT NZ): a randomized controlled trial to assess the effectiveness of a healthy lifestyle program for overweight men delivered through professional rugby clubs

**DOI:** 10.1186/s12966-022-01395-w

**Published:** 2023-03-28

**Authors:** Ralph Maddison, Elaine Anne Hargreaves, Yannan Jiang, Amanda Jane Calder, Sally Wyke, Cindy M. Gray, Kate Hunt, David Revalds Lubans, Helen Eyles, Nick Draper, Ihirangi Heke, Stephen Kara, Gerhard Sundborn, Claire Arandjus, Lan Gao, Peter Lee, Megumi Lim, Samantha Marsh

**Affiliations:** 1 National Institute for Health Innovation, University of Auckland, Auckland, New Zealand; 2 Institute for Physical Activity and Nutrition, Deakin University, Geelong, Australia; 3 School of Physical Education, Sport & Exercise Sciences, University of Otago, Dunedin, New Zealand; 4 Department of Statistics, Faculty of Science, University of Auckland, Auckland, New Zealand; 5 Institute of Health and Wellbeing, College of Social Sciences, University of Glasgow, Glasgow, Scotland; 6 Institute for Social Marketing and Health, Faculty of Health and Sports Sciences, University of Stirling, Stirling, Scotland; 7 School of Education, Centre for Active Living and Learning, University of Newcastle, Hunter Medical Research Institute, Newcastle, Australia; 8 Department of Epidemiology and Biostatistics, National Institute for Health Innovation, University of Auckland, Auckland, New Zealand; 9 Faculty of Health, SHARRC, University of Canterbury, Christchurch, New Zealand; 10 Heke Consulting, Auckland, New Zealand; 11 Axis Sport Medicine Clinic, Auckland, New Zealand; 12 Department of Pacific Health, University of Auckland, Auckland, New Zealand; 13 Deakin Health Economics, Institute for Health Transformation, School of Health and Social Development, Faculty of Health, Deakin University, Geelong, Australia

**Keywords:** Physical activity, Obesity, Weight loss, Men’s health, Lifestyle intervention

## Abstract

**Background:**

A healthy lifestyle program that appeals to, and supports, overweight and obese New Zealand (NZ) European, Māori (indigenous) and Pasifika men to achieve weight loss is urgently needed. A pilot program inspired by the successful Football Fans in Training program but delivered via professional rugby clubs in NZ (*n* = 96) was shown to be effective in weight loss, adherence to healthy lifestyle behaviors, and cardiorespiratory fitness in overweight and obese men. A full effectiveness trial is now needed.

**Aims:**

To determine the effectiveness and cost effectiveness of Rugby Fans In Training-NZ (RUFIT-NZ) on weight loss, fitness, blood pressure, lifestyle change, and health related quality of life (HRQoL) at 12- and 52-weeks.

**Methods:**

We conducted a pragmatic, two-arm, multi-center, randomized controlled trial in NZ with 378 (target 308) overweight and obese men aged 30–65 years, randomized to an intervention group or wait-list control group. The 12-week RUFIT-NZ program was a gender-sensitised, healthy lifestyle intervention delivered through professional rugby clubs. Each intervention session included: i) a 1-h workshop-based education component focused on nutrition, physical activity, sleep, sedentary behavior, and learning evidence-based behavior change strategies for sustaining a healthier lifestyle; and 2) a 1-h group-based, but individually tailored, exercise training session. The control group were offered RUFIT-NZ after 52-weeks. The primary outcome was change in body weight from baseline to 52-weeks. Secondary outcomes included change in body weight at 12-weeks, waist circumference, blood pressure, fitness (cardiorespiratory and musculoskeletal), lifestyle behaviors (leisure-time physical activity, sleep, smoking status, and alcohol and dietary quality), and health-related quality of life at 12- and 52-weeks.

**Results:**

Our final analysis included 200 participants (intervention *n* = 103; control *n* = 97) who were able to complete the RUFIT-NZ intervention prior to COVID-19 restrictions. At 52-weeks, the adjusted mean group difference in weight change (primary outcome) was -2.77 kg (95% CI -4.92 to -0.61), which favored the intervention group. The intervention also resulted in favorable significant differences in weight change and fruit and vegetable consumption at 12-weeks; and waist circumference, fitness outcomes, physical activity levels, and health-related quality of life at both 12 and 52 weeks. No significant intervention effects were observed for blood pressure, or sleep. Incremental cost-effective ratios estimated were $259 per kg lost, or $40,269 per quality adjusted life year (QALY) gained.

**Conclusion:**

RUFIT-NZ resulted in sustained positive changes in weight, waist circumference, physical fitness, self-reported physical activity, selected dietary outcomes, and health-related quality of life in overweight/obese men. As such, the program should be recommended for sustained delivery beyond this trial, involving other rugby clubs across NZ.

**Trial registration:**

Australia New Zealand Clinical Trials Registry, ACTRN12619000069156. Registered 18 January 2019, https://www.anzctr.org.au/Trial/Registration/TrialReview.aspx?id=376740 Universal Trial Number, U1111-1245–0645.

**Supplementary Information:**

The online version contains supplementary material available at 10.1186/s12966-022-01395-w.

## Background

In New Zealand (NZ), 31% of adults are obese (BMI > 30 kg/m^2^) and a further 35% are overweight, with key sex and ethnic disparities. Compared with women, the prevalence of overweight is greater in NZ European (41% vs 32%), Māori (the indigenous peoples of NZ; 33% vs 27%), and Pasifika (26% vs 16%, respectively) men [1]. Further, Māori and Pasifika men are 1.7 and 2.2 times more likely to be obese when compared with non-Māori and non-Pasifika men, respectively. It has been estimated that excess weight costs NZ at least $NZ 2 Billion per year [2]. Therefore, effective, sustainable and appealing healthy lifestyle programs are needed to meet the needs of overweight and obese NZ men, and in particular Māori and Pasifika men [3, 4].

Men are underrepresented in obesity services, suggesting current weight loss services are suboptimal for this group [5]. A systematic review and meta-analysis of men-only weight loss and weight maintenance programs, involving 14 randomized controlled trials (RCTs) showed that the most effective interventions combined dietary, exercise, and behavior change techniques (mean difference in weight at 1 year compared with no intervention was -4.9 kg, 95% confidence interval -5.9 to -4.0, *p* < 0.0001). Group-based interventions also produced favorable weight loss results [6].

Building on the successful Football Fans In Training (FFIT) program, a weight management and healthy lifestyle program in Scotland [7, 8], we previously developed and piloted a similar program to support overweight/obese men to lose weight, delivered via professional rugby clubs in NZ. In our pilot RCT (*N* = 96) of Rugby Fans In Training-NZ (RUFIT-NZ) we found a -2.5 kg (95% CI -0.4 to 5.4) difference in body weight in favor of participants in the intervention group at 12-weeks. In addition, participants who received the program had significant reductions in waist circumference, resting heart rate, and diastolic blood pressure, as well as improved fitness and adherence to lifestyle behaviors, including physical activity and not smoking [9]. Furthermore, 100% of those who completed the program said that they would recommend it to their friends, and qualitative data from a subset of men found that the factors incorporated into the design and delivery of the program created engagement [10]. Therefore the feasibility and acceptability of RUFIT-NZ was demonstrated, supporting the need for a larger scale RCT to evaluate its sustained effect [9]. The aim of the current study was to determine the effectiveness and cost effectiveness of RUFIT-NZ on weight loss, fitness, blood pressure, lifestyle change, and health related quality of life (HRQoL) at 12- and 52-weeks.

## Methods

### Study design

A pragmatic multi-center, two-arm, parallel RCT was conducted in NZ between 21 Jan 2019 and 22 Oct 2020. The study received ethical approval from the University of Auckland Human Ethics Committee (021,888). The study protocol was registered and published [11] before the conclusion of recruitment (Australian New Zealand Clinical Trials Registry, ID: ACTRN12619000069156 Registered, 18 Jan 2019). The trial was designed and reported according to the Consolidated Standards of Reporting Trials (CONSORT) checklist [12, 13] (Additional file 1). Some changes to study methods were implemented as a result of COVID-19 (see details below).

### Study setting

RUFIT-NZ was delivered via professional rugby franchises, which participate in the Super Rugby competition across NZ, Australia and South Africa. The five NZ-based Super Rugby franchises were approached and three agreed to participate (the Blues based in Auckland [North Island], Crusaders based in Christchurch, and Highlanders based in Dunedin [both South Island]).

### Participants and recruitment

Eligible participants were overweight men (defined as a BMI ≥ 28 kg/m^2^) aged 30–65 years, who were able to safely undertake physical activity, understand and read English, and provide written informed consent. Interested and eligible participants were pre-screened using the Physical Activity Readiness Questionnaire (PAR-Q) [14, 15], and required consent from their general practitioner if any PAR-Q items were endorsed. Exclusion criteria included participation in any other healthy lifestyle program, or if participants knew in advance they could not complete the 52-week follow-up. Participants were recruited via the respective rugby club’s fan base registries, including Facebook pages, supporter mailing lists, and newspaper advertisements/articles. Participants were also recruited via Māori-specific networks (e.g., Marae [Māori meeting house], word-of-mouth) and media (e.g., Māori television and radio). We linked all advertisements with the University of Auckland’s Faculty of Medical and Health Science’s research study recruitment page where participants could access information about the study, including the Participant Information Sheet and Consent Form. There was also the option to contact the research team for additional information as required, and potential participants could link directly to an online registration form. Our multifaceted recruitment strategy was informed by the RUFIT-NZ pilot study, which suggested that club-based and social media strategies were likely to be most effective. An example of a recruitment flyer is provided (see supplementary file).

### Sample size

A total of 308 participants (154 per arm) was estimated to provide 90% power at 5% significance level (two-sided) to detect a clinically significant 5 kg difference [16] on the primary outcome (change in weight) between the two groups at 52-weeks, assuming a standard deviation (SD) of 12 kg and allowing for 20% loss to follow up. Our SD was conservative and derived from other weight management trials for men [9, 17]. Māori are the indigenous population of NZ, therefore we wanted to ensure sufficient power to detect effects for this group. We therefore aimed to recruit a total of 150 Māori participants (~ 50% of the total sample size), which was estimated to provide 80% power to detect a 6 kg difference between ethnic groups under the same assumptions.

### Randomization

Following baseline data collection, eligible and consented participants were randomized on a 1:1 ratio to either the RUFIT-NZ intervention or the control group using a computerized randomization process that ensured allocation concealment. Randomization was stratified by baseline BMI category (< 35 kg/m2 versus ≥ 35 kg/m2), self-reported ethnicity (Māori, Pasifika, non-Māori/non-Pasifika), and study center, using stratified block randomization with variable block sizes of two or four. The randomization sequence was generated by our biostatistician (YJ). Participants were informed by email of their eligibility and allocation group within 2–3 days of their baseline assessment at the club. Due to the nature of the study, participants and research assistants were aware of the treatment allocation post-randomization. Study investigators and the trial statistician were blinded during analysis. To reduce assessment bias, objective measures of height and weight were collected by trained researchers at 12- and 52-weeks.

### Control and intervention

#### Control group

We used a wait-list control approach— those randomized to the control group were asked to continue with their usual lifestyle for 52 weeks during the trial period but were offered the RUFIT-NZ intervention at the end of the 12-month follow-up period.

#### Intervention

Full details of the development of the RUFIT-NZ intervention are published in our study protocol [11]. The overall aim of the intervention was to support men to engage in healthy lifestyle behaviors to reduce weight and develop the necessary skills to maintain these behaviors in the long-term. RUFIT-NZ involved a 12-week healthy lifestyle program, consisting of 12 × weekly 2-h sessions. Each intervention session included a 1-h workshop-based education component (See Appendix) and 1-h group-based, but individually tailored, exercise training session. During the education component, participants were introduced to a range of topics relating to physical activity, nutrition, sleep, and alcohol consumption, as well as to key theory-based behavior change techniques.

Education sessions were delivered predominantly by RUFIT-NZ-trained trainers, however nutrition-based components were delivered by the clubs’ nutritionists or qualified dieticians, supported by the study nutritionist (HE). This approach differed from the original FFIT program but was consistent with the RUFIT-NZ pilot, which indicated a preference for expert advice on diet and nutrition. All RUFIT-NZ trainers were qualified strength and conditioning trainers involved with the respective rugby clubs. Registered dietitians involved in delivering RUFIT-NZ had a previous connection to the club. For the purpose of this trial, the trainers and nutritionists were employed by the respective clubs and agreed to deliver RUFIT-NZ. Classroom content was standardized, so that all participants received the same education information, but the individual trainers could tailor the format of delivery and level of detail as required. RUFIT-NZ did not engage professional team players in the delivery of the intervention. That decision was based on previous experience with FFIT [7] and our previous pilot trial [9]. The education sessions and the overall delivery of the program was interactive, with RUFIT-NZ trainers and dieticians enabling interactive learning and encouraging camaraderie and a sense of team to facilitate discussion of key topics.

Group-based in-stadia physical activity sessions were delivered by the trainers who were given basic guidance to deliver sessions (e.g., start low and build slow), but were also given freedom to structure each session as they chose. This approach allowed trainers to best meet the needs of individuals attending their RUFIT-NZ sessions. Activity sessions were tailored to individual fitness levels and ability. They included aerobic (e.g., stationary rowing and cycling, walking and jogging), muscle strengthening (e.g., weight/circuit training) and flexibility (e.g., warm-up/cool-down activities) exercises [18]. Participants were instructed to use the rating of perceived exertion (RPE) scale to ensure their activity was appropriate for their own fitness level. The difficulty (intensity) of each physical activity session increased over the 12 weeks, accounting for each participant’s level of fitness. Throughout the intervention men were encouraged to consider what types of activity they could continue to engage with in community settings. Sessions were varied and utilized the supportive group involvement to foster the sense of being in a ‘team’. Group size ranged from approximately 15–20 men per trainer. Roll calls were taken at the beginning of each session to record attendance.

To inspire habitual physical activity, men were encouraged to follow a daily step-based walking program over the course of the 12-week intervention period and beyond [19–21] and to use a step counter (pedometer or smartphone app) to track their daily and weekly progress. Trainers encouraged men to engage in other forms of physical activity and with a focus on integrating walking and other forms of incidental activity into daily life (e.g., walking up stairs). RUFIT-NZ trainers also provided physical activity ‘homework’ that participants could undertake outside of the structured sessions (e.g., researching places in their community to be physically active). Participants’ lifestyle behaviors in terms of alcohol, sleep, sedentary behavior, and nutrition were guided by individual goals, which men set for themselves during the group education sessions and recorded in a workbook.

Nutrition content for RUFIT-NZ was developed by our investigator nutritionist (HE), and was consistent with the NZ guidelines approach for weight management using a Family, Activity, Behavior (FAB) approach [22]. Our aim was to ensure all nutrition sessions were positively framed (e.g., ‘what are some good examples of healthy snacks?’ and ‘where can I find quick easy recipes?’), and involved the delivery of simple messages focused on practical elements of improving diet. Messages aligned with the NZ Eating and Activity Guidelines for Adults [23]. To facilitate an understanding of what men were eating and to help them record their diet, we provided men with a food diary to use as they wished. RUFIT-NZ nutrition sessions targeted the following biggest healthy eating ‘wins’:

- Eating as many fruit and vegetables as possible.
- Cooking and preparing food and snacks at home as much as possible.
- Eating mostly whole foods (as opposed to packaged/processed foods and takeaways).
- Drinking sugar-free beverages.
- Conscious eating (screen-free, mindful eating, ideally in the company of others).

### Behavior change techniques

A key focus of RUFIT-NZ was to provide men with a range of skills and strategies they could use to develop and maintain a healthy lifestyle, which included managing their weight. To that end, a range of evidence-based behavior change techniques shown to be effective in improving diet and physical activity were used throughout the education and exercise sessions [24]. Key techniques included: i) identifying autonomous reasons for lifestyle change, ii) goal setting for, and self-monitoring of, weight, physical activity, and healthy diet; iii) intention formation with action plans; iv) experiencing exercise sessions with increased challenges as well as positive feedback on exercise achievements and change reinforcement from trainers to build self-efficacy; and v) identification of barriers and coping planning to help avoid relapse during, and on completion of, the program (see Appendix, for details).

### Training

Prior to delivering RUFIT-NZ, trainers underwent a standardized training session, delivered by a member of the RUFIT-NZ investigator team. Training was supplemented with a standardized trainer’s manual, which outlined key principles to be promoted via RUFIT-NZ, and the nutrition topics to be covered in each session. We also provided PowerPoint presentation templates and participant worksheets, and support sessions and resources were offered to nutritionists to use in group-based sessions. Trainers were provided a nominal fee (paid to the club) to deliver RUFIT-NZ.

For RUFIT-NZ we used the Supportive, Active, Autonomous, Fair, and Enjoyable (SAAFE) delivery principles, an evidence-based approach for the planning, delivery, and evaluation of organized physical activity sessions [25]. Consistent with this approach, trainers were encouraged to ensure a SAAFE environment by: i) creating a Supportive social environment, enabling learning from each other; ii) maximizing participants’ opportunities to be physically Active during the sessions; iii) satisfying participants’ need for Autonomy by including elements of choice and providing a rationale for activities; iv) designing and delivering activities that are Fair by allowing all participants to experience success regardless of their physical abilities; and v) promoting an Enjoyable experience by focusing on fun and variety and incorporating games where possible.

### Fidelity

To assess intervention fidelity, we undertook direct observation by trained research assistants, using a standardized checklist at Weeks 4 and 10 of the 12-week intervention. Components of the classroom sessions (e.g., attendance, correct slides, weight check, and delivery) and the physical activity sessions (e.g., individualized exercises, adherence to SAAFE principles, and general delivery) were assessed on whether they were delivered or not; the research team provided verbal and written feedback to the trainers to address any gaps in intervention delivery. All coaches passed these checks.

### Outcomes

All outcomes were assessed at 12- and 52-weeks post-randomization. The primary outcome was change in body weight from baseline to 52-weeks.

Secondary outcomes included change in body weight at 12-weeks, waist circumference, blood pressure, fitness, lifestyle behaviors and other nutrition outcomes, health-related quality of life (HRQoL) and cost-effectiveness.

*Anthropometric data* were collected using standard practices [26]. Height was measured to the nearest 0.1 cm with a stadiometer (Seca, 214, Hamburg, Germany) and weight was measured to the nearest 0.1 kg with a digital scale (Tanita, UM-070, Illinois, US). For both height and weight, two measures were taken. A third measurement was taken if differences of 0.1 cm and 0.1 kg respectively were observed between the first and second measurements. The mean of two measurements or the median of three was used for analysis.

*Resting systolic and diastolic blood pressure* were measured via standard procedures using an automated sphygmomanometer (OMRON T9P Intellisense Blood Pressure Monitor) and/or a manual Blood Pressure Monitor (D1537-Reister Shock Proof Sphygmomanometer). Participants were asked to rest for a period of 5-min before measurements were taken.

*Cardiorespiratory fitness* was assessed using the time to complete a 6 km cycle test using a stationary bike [27].

*Musculoskeletal fitness* (endurance) was assessed using the timed sit-to-stand test [28], and a timed push up test [27].

### Lifestyle behaviors

Participants self-reported the following lifestyle behaviors, (1) leisure-time physical activity (assessed by the Godin Leisure Time Physical Activity Questionnaire) [29]; (2) cigarette smoking (assessed by a smoking history questionnaire) [30]; (3) alcohol intake (assessed by the Alcohol Use Disorders Identification test consumption [AUDIT C]) [31]; (4) sleep (self-reported average number of hours slept over a 24 h period); and nutrition habits over the past seven days, including average fruit and vegetable intake over the past week (with options from ‘I don’t eat fruit or vegetables’ to ‘4 or more servings per day), fast food or takeaway consumption (number of times in the past seven days), and sugar-sweetened beverage consumption (powdered and fizzy drinks; number of times over the past seven days). All nutrition habits questions were assessed using existing questions from the NZ Health Survey [32]. The Godin Leisure Time Questionnaire has been shown to correlate with maximal oxygen consumption (r = 0.24 -0.34) [33], and accelerometry (r = 0.32–0.45) (cf., [34]). Cigarette smoking questions were adapted from the Fagerström Test for Nicotine Dependence, which has demonstrated significant mean differences in the number of cigarettes smoked and Carbon Monoxide measures [35]. The AUDIT-C is an abbreviated version of the Alcohol Use Disorders Identification test (AUDIT) that has been advocated for use in both research and practice settings where there is insufficient time to administer the full AUDIT [36]. The AUDIT-C has similar accuracy to the full AUDIT for providing cut-off scores for units of alcohol consumed [37]; cut-off scores are based on expert opinion rather than validation data. No validation data were available for sleep or the fruit and vegetable questions, however we utilized the standardized questions outlined in the 2006/07 NZ Health Survey.

### Other dietary factors

In addition to fruit and vegetable consumption, we collected data on meals consumed or prepared at home (number of times for breakfast, lunch and dinner over the past week), and conscious eating (noticing when eating and not hungry, and stopping eating when full (scale from one to four, with four ‘agree’); these questions were sourced from the Framson mindful eating questionnaire and the Clementi abbreviated mindful eating questionnaire, adapted to seven days of measurement [38, 39].

Baseline demographics: Participants completed a web-based questionnaire for demographic information including age, date of birth, ethnicity, employment status, highest level of education, marital status, and household income.

## Economic evaluation

A trial-based economic evaluation was undertaken using data from the RUFIT-NZ trial. The main outcomes for the economic evaluation were the incremental cost-effectiveness ratio (ICER) in terms of cost per body weight loss (kg), and cost per quality-adjusted life year (QALY) gained, for participants enrolled in the program compared with those in the control arm over 52 weeks. Participant HRQoL was measured using the EQ-5D-5L at both 12 and 52 weeks; a utility score was derived using NZ population-specific weights to estimate QALYs gained at 52 weeks for participants enrolled in both treatment and control arms [40, 41]. A minor protocol violation meant that the EQ5-D was not administered at baseline.

## Changes in response to the COVID-19 Pandemic

The present study was severely affected by the COVID-19 pandemic and changes to our original trial are detailed below. For logistical reasons, participants were recruited for the RUFIT-NZ trial in three separate waves. Prior to COVID-19 we successfully recruited and randomized 200 participants over two waves (2019). Participants for wave three were recruited and randomized in February 2020, but due to strict COVID-19 restrictions and lockdowns across NZ, as well as changes in health and safety requirements at the respective rugby clubs, we were unable to continue with wave three. As a result, none of the participants randomized (intervention or control) in wave three (*n* = 178) took part in the RUFIT-NZ program. As a result, the steering group committee made the decision to only analyze data from the 200 participants randomized to waves 1 and 2. A post-hoc power evaluation on the study sample suggested that we had > 90% power to detect a group difference of 3 kg on weight change at 52 weeks. Therefore, in the results section we only report findings on those 200 participants; baseline data from all participants are presented in the Appendices.

## Analysis

Trial data from all randomized participants were collected via secure web-based case record forms and stored using REDCap. Baseline characteristics and outcome data were first summarized descriptively: continuous variables were summarized as mean and standard deviation (SD), and categorical variables as frequency and percentage.

Analysis was conducted according to a modified intention-to-treat (ITT) principle, to include all randomized participants in the first two waves. For the main ITT analysis, we used multiple imputations for missing primary outcome data. Sensitivity analysis was also considered using the baseline value carried forward (BVCF) approach on missing data to test the robustness of the main results. Per protocol (PP) analysis was also performed on randomized participants who provided primary outcome data with no major protocol violations. Linear regression models were used to evaluate the effect of the intervention on change in body weight at 52 weeks (primary outcome), adjusting for baseline body weight, age, study wave, and stratification factors (BMI category, self-reported ethnicity, study center). Given the COVID-19 related issues, we were unable to undertake sub-group analysis by ethnicity.

Similar regression models were used on continuous secondary outcomes at 12- and 52-weeks. Model-adjusted mean differences were reported with 95% confidence intervals (CIs). Logistic regression models were used on the adherence to healthy lifestyle behaviors outcomes at 12 and 52 weeks. Adjusted odds ratios (ORs) were reported with 95% CIs. For secondary outcomes, no imputation was considered on missing outcome data. Confidence interval widths have not been adjusted for multiplicity and may not be used in place of hypothesis testing. Statistical analysis was performed using SAS version 9.4 (SAS Institute Inc.). All statistical tests were two-sided with a 5% significance level.

## Economic analysis

Multiple imputations were applied to missing primary outcome data (change in body weight, and quality of life at 52 weeks) assuming missing at random. Patterns in missing data were explored with the use of logistic regression and t-tests to investigate if any covariates would predict if an outcome variable was missing (logistic regression), and whether there were differences in patient characteristics between patient groups who had a missing outcome variable, and patient groups with a non-missing outcome variable (t-tests). To address the negatively skewed distribution for the estimated utilities, univariable generalized linear regression modelling with a Poisson distribution and log link was used to explore differences in participant QALYs following the modified Park test [41]. As with the main analysis, this model was adjusted for baseline weight, age in years, study wave, and stratification factors (BMI category, self-reported ethnicity, and study centre).

Nonparametric bootstrapping with 2,000 simulations was performed around each ICER to address uncertainty around the cost-effectiveness of RUFIT-NZ. Additionally, scenario analyses exploring the cost-effectiveness of RUFIT-NZ were performed for participants with non-missing outcomes data without imputation. The cost of providing RUFIT-NZ across the three rugby clubs was estimated through micro costing (see Appendices). Although no official willingness-to-pay threshold has been established for NZ, contemporary economic analyses have considered a gross domestic product per capita expenditure of $45,000 per QALY to be cost-effective [42, 43]. All costs were expressed in $NZD in 2021 using the consumer price index (CPI) [44].

## Results

### Overview

**Fig. 1:**
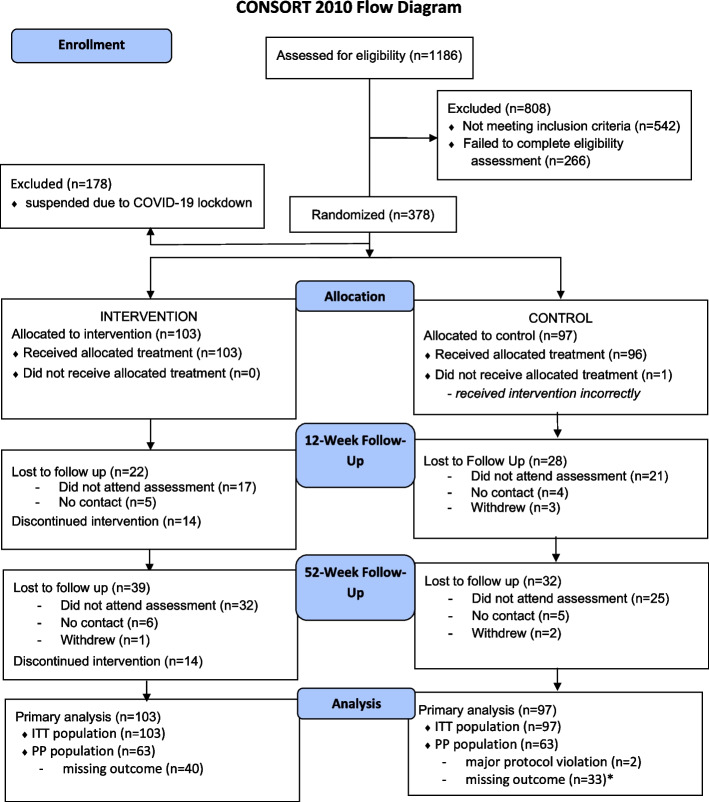
CONSORT 2010 Flow Diagram. Note: ITT – Intention to treat; PP – Per protocol. *One participant was randomized to the wrong group but also had missing primary outcome as well and was therefore reported in the missing outcome numbers

Figure 1 presents the flow diagram of participant progress through the phases of the trial. A total of 1,186 people were screened between January 2019 and February 2020, and 378 eligible participants were randomized (Intervention *n* = 192; Control *n* = 186). The final analysis included a total of 200 trial participants (Intervention *n* = 103; Control *n* = 97) who were able to complete the study in waves one and two, prior to COVID-19 restrictions. No baseline differences were found between the participants of different waves (see Appendices).

Two participants had a major protocol violation after randomization and were excluded from analysis. One participant had a baseline BMI below the < 28 kg/m^2^ cutoff in the inclusion criteria. Another participant was randomized to the control group but allocated to the intervention group in error. The participant was informed of the mistake but continued with the intervention. As highlighted above, a separate minor protocol violation was recorded as the EQ5-D was not administered at baseline.

Participants were predominantly New Zealand European, with a mean age of 45.7 years (SD 8.7) years. The two groups were comparable at baseline (Table 1).

**Table 1:** Participant baseline demographic and study site data (*N* = 200)

Baseline Characteristics		Control (*n* = 97)		Intervention (*n* = 103)
**Demographics**				
Age (years), mean (SD)		46.3 (8.7)		45.1 (8.7)
**Ethnicity, n (%)**				
Māori		16 (16.5)		21 (20.4)
Pasifika		16 (16.5)		16 (15.5)
New Zealand European or Other		65 (67.0)		66 (64.1)
**BMI category, n (%)**				
28–34 kg/m^2^		53 (54.6)		56 (54.4)
≥ 35 kg/m^2^		44 (45.4)		47 (45.6)
**Annual household income, n (%)**				
Less than $NZD15,000		1 (1.0)		1 (1.0)
$NZD15,000—$29,999		0 (0.0)		4 (3.9)
$30,000—$59,999		9 (9.3)		14 (13.6)
$60,000—$99,999		32 (33.0)		30 (29.1)
$100,000 or more		49 (50.5)		46 (44.7)
Did not know / chose not to answer		6 (6.2)		8 (7.8)
**Marital status, n (%)**				
Married		64 (66.0)		72 (69.9)
Civil union or living with partner		22 (22.7)		14 (13.6)
Separated, divorced, or widowed		9 (9.3)		7 (6.8)
Never married (single)		2 (2.1)		5 (4.9)
Refuse to answer		0 (0.0)		5 (4.9)
**Study wave, n (%)**				
Wave 1 (Feb 2019 – Feb 2020)	56 (57.7)		55 (53.4)	
Wave 2 (May 2019 – Jun 2020)	41 (42.3)		48 (46.6)	
**Study site, n (%)**				
Blues	48 (49.5)		52 (50.5)	
Crusaders	21 (21.6)		23 (22.3)	
Highlanders	28 (28.9)		28 (27.2)	

Note: Data presented from participants recruited in waves one and two only

### Effect on body weight and secondary outcomes

Participants in the control group showed little change in weight from baseline to 12-weeks (mean = -0.38, SD 4.82 kg) and 52-weeks (mean = 0.10, SD 5.73 kg), while the intervention group demonstrated weight losses from baseline at both time points (12-weeks: mean -2.65, SD 3.42 kg; 52-weeks: mean -2.59, SD 6.95 kg). In the primary ITT analysis with multiple imputations on missing outcome, the adjusted mean difference in weight change between the two groups was -2.77 kg (95%CI -4.92 to -0.61) in favour of the intervention group (Table 2). In the per-protocol analysis, the adjusted mean difference in weight change between the two groups was -2.82 kg (95%CI -4.99 to -0.65), also in favour of the intervention group. The sensitivity analysis using the BVCF approach on missing outcome gave an estimated group difference of -1.95 kg (95%CI -3.34 to -0.56).

**Table 2:** Primary outcome analysis on change in body weight at 52 weeks

	Control	Intervention	Intervention vs Control^*^	
	N, Mean (SD)	N, Mean (SD)	Adjusted Mean difference (95% CI)	*p*-value
Change from baseline	64, 0.10 (5.73)	63, -2.59 (6.95)	-	-
ITT with MI (primary)	-	-	-2.77 (-4.92, -0.61)	0.015
ITT with BVCF	-	-	-1.95 (-3.34, -0.56)	0.006
PP	-	-	-2.82 (-4.99, -0.65)	0.011

*N* numbers observed, *SD* standard deviation, *ITT* intention to treat, *MI* multiple imputations, *BVCF* baseline value carry forward, *PP* per protocol

^*^Linear regression model adjusted for baseline weight, age in years, study wave, and stratification factors (BMI category, self-reported ethnicity, study centre)

For secondary outcomes reported in Table 3, a similar mean difference in weight change was observed at 12-weeks (-2.65 kg; 95%CI -3.90 to -1.41) in favour of the intervention group. There was a significant difference in waist circumference at both time points in favour of the intervention. There were no between group differences in blood pressure at either time point. There were significant differences in cardiorespiratory fitness, sit to stand times, and press-ups completed, all favouring the intervention group. In terms of lifestyle change, there were positive differences in self-reported physical activity at 12- and 52-weeks, and fruit and vegetable consumption at 12 weeks (with some attenuation at 52 weeks), but no significant differences in number of alcoholic drinks consumed per week, or hours of sleep per day at both time points. Significant differences were observed in HRQoL at both 12- and 52 weeks in favour of the intervention group (Table 3).

**Table 3:** Secondary outcome analysis on anthropometric data, blood pressure, fitness, lifestyle behaviors and HRQoL at 12- and 52-weeks

Outcomes		Control		Intervention		Intervention vs Control^*^			
		N	Mean (SD)	N	Mean (SD)	Mean difference	95% CI		*P* value
Weight (kg)	Baseline	97	111.46 (17.25)	103	112.13 (19.35)				
	12-weeks	69	112.52 (17.93)	81	107.85 (18.26)	-2.65	(-3.90, -1.41)		< .0001
	52-weeks	64	111.34 (19.76)	63	108.47 (19.86)	-2.82	(-4.99, -0.65)		0.011
BMI (kg/m^2^)	Baseline	97	35.30 (4.87)	103	35.55 (5.65)				
	12-weeks	67	35.46 (4.96)	80	34.38 (5.09)	-0.88	(-1.27, -0.48)		< .0001
	52-weeks	61	35.47 (5.48)	58	34.50 (5.52)	-0.69	(-1.42, 0.04)		0.062
Waist circumference (cm)	Baseline	97	116.93 (11.39)	103	118.14 (13.60)				
	12-weeks	67	115.55 (11.40)	80	112.48 (12.91)	-2.51	(-4.27, -0.75)		0.006
	52-weeks	61	116.29 (12.79)	58	113.00 (13.65)	-2.91	(-5.14, -0.67)		0.011
Systolic BP (mmHg)	Baseline	97	147.28 (14.19)	103	145.99 (15.91)				
	12-weeks	67	145.20 (15.71)	80	145.81 (17.54)	1.94	(-1.92, 5.80)		0.322
	52-weeks	61	147.70 (15.28)	58	143.47 (15.22)	-2.06	(-6.13, 2.01)		0.318
Diastolic BP (mmHg)	Baseline	97	93.36 (9.23)	103	91.59 (8.66)				
	12-weeks	67	90.01 (10.18)	80	90.09 (9.74)	1.57	(-0.87, 4.00)		0.205
	52-weeks	61	93.08 (9.77)	58	89.94 (10.48)	-1.63	(-4.54, 1.28)		0.270
Sit to stand test	Baseline	97	15.37 (2.84)	103	15.69 (3.23)				
	12-weeks	67	16.37 (3.64)	80	20.23 (4.36)	3.26	(2.15, 4.36)		< .0001
	52-weeks	55	15.64 (3.26)	51	19.33 (4.79)	2.73	(1.55, 3.92)		< .0001
Press up test (count)	Baseline	97	19.10 (9.37)	103	19.77 (9.53)				
	12-weeks	67	19.83 (11.39)	80	26.43 (10.40)	5.05	(2.56, 7.54)		0.0001
	52-weeks	54	19.20 (11.33)	50	24.48 (12.64)	5.09	(2.08, 8.09)		0.0011
Fitness test (mins)	Baseline	97	10.17 (1.00)	102	10.19 (0.95)				
	12-weeks	67	10.09 (0.92)	77	9.71 (0.76)	-0.37	(-0.53, -0.21)		< .0001
	52-weeks	55	10.15(1.08)	50	9.74 (0.99)	-0.48	(-0.77, -0.18)		0.0017
Sleep (hours/day)	Baseline	97	7.00 (0.92)	103	6.96 (1.00)				
	12-weeks	67	7.19 (0.94)	81	7.14 (1.20)	0.09	(-0.17, 0.34)		0.508
	52-weeks	61	6.90 (0.89)	54	6.91 (1.00)	0.09	(-0.17, 0.35)		0.489
Physical activity score	Baseline	97	29.03 (25.18)	103	28.51 (31.46)				
	12-weeks	69	36.36 (32.25)	81	50.79 (25.52)	17.10	(8.29, 25.91)		0.0002
	52-weeks	62	33.65 (19.35)	54	47.24 (25.94)	15.03	(6.98, 23.07)		0.0003
Alcohol drinks per week	Baseline	97	6.77 (8.36)	103	6.63 (7.25)				
	12-weeks	69	5.91 (7.66)	81	5.19 (6.91)	0.04	(-1.51, 1.59)		0.957
	52-weeks	62	6.05 (6.09)	54	5.52 (5.69)	-0.32	(-1.81—1.17)		0.669
Fruit and vegetable servings per day	Baseline	96	3.22 (1.64)	103	3.29 (1.54)				
	12-weeks	66	3.45 (1.71)	80	4.04 (1.53)	0.63	(0.19, 1.07)		0.005
	52-weeks	61	3.23 (1.56)	54	3.75 (1.66)	0.41	(-0.07, 0.90)		0.093
HRQoL	Baseline (not collected)								
	12-weeks	29	63.24 (19.33)	37	74.35 (13.60)	11.73	(3.65, 19.82)		0.005
	52-weeks	61	63.48 (18.34)	54	66.87 (19.42)	6.55	(0.05, 13.05)		0.048

Note: *BP* Blood pressure, *HRQoL* Health related quality of life scale measured via EQ5D (0–100, higher is better)

Secondary analysis with no imputation for missing outcome data; two participants with major protocol violations were excluded

^*^ Linear regression model adjusted for baseline outcome, age in years, study wave, and stratification factors (BMI category, self-reported ethnicity, study centre)

In terms of additional dietary outcomes, we observed favourable differences for the intervention group in the number of fizzy drinks consumed at 12 weeks, number of powdered drinks at 52 weeks, and number of fast-food occasions at 12 weeks only (Table 4). There were positive effects on the ‘stop eating when full’ score at 12 and 52 weeks, but not on the ‘noticed when eating and hunger’ score.

**Table 4:** Secondary analysis on other dietary outcomes measured at 12- and 52-weeks

Outcomes		Control		Intervention		Intervention vs Control^*^			
		N	Mean (SD)	N	Mean (SD)	Mean difference	95% CI		*P* value
Number of fizzy drinks in past week	Baseline	97	3.79 (4.04)	103	4.0 (6.04)				
	Post program	67	2.72 (3.37)	81	1.90 (3.12)	-0.81	(-1.66, 0.04)		0.06
	52-weeks	61	2.26 (3.61)	54	2.65 (4.48)	0.22	(-0.72, 1.16)		0.64
Number of powdered drinks in past week	Baseline	97	1.12 (2.32)	103	0.65 (1.68)				
	Post program	67	0.60 (1.97)	81	0.25 (0.68)	-0.16	(-0.57, 0.25)		0.43
	52-weeks	61	1.07 (2.48)	54	0.33 (1.03)	-0.69	(-1.42, 0.04)		0.06
Number of fast food occasions	Baseline	97	2.64 (2.19)	103	2.77 (2.20)				
	Post program	67	2.19 (2.15)	81	1.32 (1.16)	-1.05	(-1.51, -0.59)		< 0.0001
	52-weeks	61	1.74 (1.70)	54	1.70 (1.78)	-0.18	(-0.75, 0.40)		0.54
Noticed when eating and hunger score	Baseline	97	1.99 (0.73)	103	2.10 (0.71)				
	Post program	67	1.91 (0.57)	81	1.94 (0.81)	-0.01	(-0.24, 0.22)		0.91
	52-weeks	61	1.97 (0.68)	54	1.87 (0.85)	-0.13	(-0.42, 0.16)		0.36
Stop eating when full score	Baseline	97	1.89 (0.81)	103	2.02 (0.91)				
	Post program	67	2.19 (1.00)	81	2.67 (0.88)	0.39	(0.11, 0.67)		0.007
	52-weeks	61	2.02 (0.85)	54	2.50 (0.82)	0.38	(0.10, 0.67)		0.009

Note: Noticed when eating and hunger score – higher score is better. Stope eating when full score – higher is better

Secondary analysis with no imputation on missing outcome data; two participants with major protocol violations were excluded

^*^Linear regression model adjusted for baseline outcome, age in years, study wave, and stratification factors (BMI category, self-reported ethnicity, study centre)

**Cost-effectivenes﻿s:** Based on a total intervention cost of $77,469, the cost per participant was estimated to be $ NZD 752. Control participants were assumed to incur no costs (derivation of costs can be found in the Appendices).

Based on the results of non-parametric bootstrapping following multiple imputation, slight QALY gains were observed for participants in the intervention arm in bootstrapped analysis (mean QALY gains: 0.02, 95% CI: 0.01 – 0.03, P < 0.001). ICERs estimated for participants in the RUFIT-NZ intervention were $259 per kg lost, or $40,269 per QALY gained, and RUFIT-NZ was cost-effective in 78% of 2,000 iterations if a willingness-to-pay threshold of $45,000 NZD per QALY was used. In a scenario analysis of non-imputed data, the cost-effectiveness of RUFIT-NZ was maintained for the primary outcome of body weight reduction at 52 weeks, but not for QALYs gained (ICER: $231,895 per QALY gained) (Table 5).

**Table 5:** Results of the economic evaluation

Parameter	Analysis	Difference	*P*-value	Cost ^c^
		**Mean (95% CI) **^**a,b**^		
QALYs gained	Base-case	0.02 (0.01 – 0.03)	< 0.001	$752
	Non-imputed	0.01 (-0.03 – 0.06)	0.623	$752
**Cost-effectiveness analysis**		**ICER**	**Probability of cost-effectiveness (%) **^**a**^	
Cost per QALY gained	Base-case	$40,269 per QALY gained	78.0	
	Non-imputed	$231,895 per QALY gained	42.9	
Cost per kg body weight loss	Base-case	$259 per KG weight loss	-	
	Non-imputed	$252 per KG weight loss	-	

*ICER* incremental cost-effectiveness ratio, *KG* kilogram, *QALY* quality-adjusted life year

^a^ Based on 2,000 bootstrapped iterations following multiple imputation

^b^ Generalised linear regression model adjusted for baseline weight, age in years, study wave, and stratification factors (BMI category, self-reported ethnicity, study centre)

^c^ The cost of RUFIT-NZ was $752 per participant; no costs were incurred for participants in the control arm

## Discussion

The aim of our study was to determine the effectiveness of the 12-session, healthy lifestyle RUFIT-NZ program on weight loss, fitness, blood pressure, and lifestyle change at 12- and 52-weeks. Overall, our findings showed the program resulted in sustained changes in weight, waist circumference, physical fitness, self-reported physical activity, alcohol consumption, and HRQoL in overweight men. The RUFIT-NZ intervention, as assessed through cost per kg body weight loss and QALYs gained, was broadly comparable with other economic evaluations of weight loss interventions and was highly likely to be cost-effective at a willingness-to-pay threshold of $45,000 NZD per QALY [45, 46]. The modest gain in QALYs is likely attributed to the short time period (one year) of the evaluation, as the key benefits attributed to weight loss interventions lie in the prevention of downstream events of morbidity and mortality associated with obesity [45, 46]. As such, a separate cost-effectiveness analysis exploring the impact of ongoing weight reduction through RUFIT-NZ on lifetime morbidity and mortality risk is planned. The cost-effectiveness of RUFIT-NZ, in terms of cost per QALYs, was not maintained in a sensitivity analysis of non-imputed data; this is likely attributed to the high proportion of missing HRQoL data.

Strength and limitations: We conducted a pragmatic RCT based on extensive feasibility and pilot work [9]. The inclusion of three Super Rugby franchises located in different parts of the country with substantially distinct ethnic compositions (Auckland has a larger proportion of Māori and Pacific peoples compared with Dunedin) [43], enhanced the generalizability of the findings. Of the various adaptations and wider implementation of FFIT [47], RUFIT-NZ included a wider inclusion of a country’s major ethnic minority populations. Specifically, we recruited a large proportion of Māori (18%) and Pacific (16%) participants, which is representative of the respective groups’ population in NZ, and highly relevant to the NZ context given the burden of overweight and obesity in these groups.

The major limitations of this trial were related to COVID-19 disruptions. A recent paper highlighted the effect of the COVID-19 pandemic and the need to acknowledge COVID–related changes to studies [48]. Due to COVID-19 enforced lockdowns and restrictions we were unable to include the third and final wave of participants in the analyses. While we had recruited and randomized those participants, we were unable to deliver the intervention, and thus, in consultation with our trial steering committee, we made a pragmatic decision to analyze only those participants from the first two waves. Notwithstanding those issues, we had sufficient power to detect differences in the primary outcome between groups. This was a reflection of the estimated SD (12.0) used for the sample size calculation, which was larger than the observed SD (5.0) in the present study. Other limitations of our trial include the lack of blinded outcome assessments, and potential contamination, however we were unaware of any participants who were randomized to RUFIT-NZ but engaged with control participants at the same club. Finally, while we undertook extensive efforts to follow-up participants, there remained a large proportion of missing data. Findings from this trial should be interpreted with those collective limitations in mind.

The sustained weight loss (2.7 kg) at 52 weeks was slightly larger than observed in our RUFIT-NZ pilot study at 12-weeks [9], and was similar to that observed in the trial of the EuroFIT healthy lifestyle program [49] across four countries (England, Norway, Netherlands and Portugal) (mean between groups difference in weight at 12-months -2.4 kg, 95%CI -3,1 to -1.7). However, it was less than the 12-month weight loss in FFIT (mean difference in weight loss at 12-months -4.94 kg, 95% CI 3.95–5.94) [7]. Reasons for these differences are unclear. While RUFIT-NZ was inspired by FFIT, there were some differences in intervention delivery. Specifically, while trainers largely delivered education content and training sessions in RUFIT-NZ, a nutritionist delivered the nutrition content. The program was also modified to meet the cultural needs of NZ men. Despite these differences, compared with previous studies, RUFIT-NZ produced similarly positive changes in physical activity levels, fruit and vegetable consumption (but attenuated at 52-weeks), and HRQoL. However, in contrast to FFIT we did not find an effect on blood pressure.

Given the considerable burden of overweight and obesity on Māori and Pasifika men, we aimed to recruit 50% Māori. While we did not reach this target, our study was successful in recruiting large numbers of both Māori and Pasifika men. Results from our present study highlight the potential for our RUFIT-NZ to be implemented at-scale to engage these populations and to support positive lifestyle changes in a culturally acceptable way. Findings from our pilot trial demonstrated NZ men found RUFIT-NZ was acceptable [9] and features within the program (creation of a team environment, motivating coach, knowledge gained from education sessions) and those men brought to the program (motivation, support of others) created the engagement with it [10]. Combined with experience from FFIT that has shown that word-of-mouth recommendations by past participants to be one way of sustaining delivery [47], we might feasibly expect that more Māori and Pasifika men may be attracted to the program in future on the basis of the positive experience of their peers who took part in RUFIT-NZ.

## Conclusion

RUFIT-NZ resulted in sustained positive changes in weight, waist circumference, physical fitness, self-reported physical activity, selected dietary outcomes, and HRQoL in overweight men. As such, the program should be recommended for sustained delivery beyond this trial and might include other rugby clubs across NZ.

## Supplementary Information

**Additional file 1.** CONSORT checklist.

**Additional file 2.** The TIDieR checklist.

## Data Availability

Data from the RUFIT-NZ are available on request to the lead author.

## Appendices

**Table ﻿6:** RUFIT-NZ workshop content

Week	Topics/Core Messages to Cover	Target behavior	Behavior change technique addressed	Facilitator
Week 1	• Welcome & getting to know each other • Focus on lifestyle behaviors vs weight • SMART Goal setting • Team photo	Exercise/ physical activity	Goal setting and intention formation Self-monitoring Social support and encouragement	Trainer
Week 2	• Whole food philosophy including social aspects • Big wins for healthy diets • Incorporating fruit and veg • Healthy drink options • Healthy serving sizes • Reading food labels	Nutrition	Identifying autonomous reasons for lifestyle change Goal setting for, and self-monitoring of healthy diet	Nutritionist
Week 3	• Menu planning • Budgeting • Shopping • Being organized/importance of routine	Nutrition	Goal setting for, and self-monitoring of healthy diet Intention formation with action plans	Nutritionist
Week 4	• Discussion of important behavior change techniques: Autonomous motivation, building confidence, goal setting, action & coping planning, self-monitoring, social support	Exercise and physical activity	Identifying autonomous reasons for lifestyle change Goal setting Self-monitoring Social support and encouragement	Trainer
Week 5	• Focusing on your ‘circle of influence’ • Eating out Mindful eating	Nutrition	Self-monitoring, social support, goal setting with intention formation with action plans	*Nutritionist*
Week 6	• Informal session designed by trainer to meet individual needs of men in team	Physical activity	Identification of barriers and coping planning, self-monitoring and review of progress	Trainer
Week 7	• Question and Answers session	Nutrition	Identification of barriers and coping planning	Nutritionist
Week 8	• Alcohol weight-related facts • Standard drink sizes • Planning your drinking	Nutrition Alcohol	Goal setting for behavior and outcome; discrepancy between current behavior and goal; health consequences	Trainer
Week 9	• Questions and Answers session	Physical activity	Identification of barriers and coping planning, review of past progress and goal setting, social support and encouragement	Trainer
Week 10	• How sleep affects weight • How much sleep we need • Signs of sleep deprivation • Sleep hygiene tips • What is sedentary behavior • How sedentary behavior affects weight • Tips for reducing SB	Sleep and sedentary behavior	Self-monitoring of behavior and outcome of behavior, goal setting for behavior and goal setting for outcome; problem-solving	Trainer
Week 11	• Importance of enjoying physical activity for long-term maintenance • Long-term behavior change & overcoming obstacles • Planning for lifestyle change • Relapse prevention	Physical activity	Social support/encouragement; goal setting for behavior and outcome	Trainer
Week 12	• Wrap-up • Motivational talk • Team photo & Certificates	Physical activity and nutrition	Focus on past success; verbal persuasion to boost self-efficacy; goal setting for behavior and outcome; problem-solving	Trainer

**Table 7:** Participant baseline demographic data comparing all randomized participants (*N* = 378) with those who completed study (*N* = 200)

	**Total cohort**		**Wave 3**			
			**No**		**Yes**	
	**N**	**%**	**N**	**%**	**N**	**%**
	378	100.0	200	100.0	178	100.0
**Study site**						
**Blues**	196	51.9	100	50.0	96	53.9
**Crusaders**	74	19.6	44	22.0	30	16.9
**Highlanders**	108	28.6	56	28.0	52	29.2
**BMI category**						
** < 35 kg/m2**	211	55.8	109	54.5	102	57.3
** > = 35 kg/m2**	167	44.2	91	45.5	76	42.7
**Ethnicity**						
**Maori**	56	14.8	37	18.5	19	10.7
**Pacific**	63	16.7	32	16.0	31	17.4
**New Zealand European/other**	259	68.5	131	65.5	128	71.9
**Marital Status**						
**Married**	247	65.3	136	68.0	111	62.4
**Civil union/living with partner**	62	16.4	36	18.0	26	14.6
**Divorced, separated or widowed**	35	9.3	16	8.0	19	10.7
**Never married (single)**	25	6.6	7	3.5	18	10.1
**Choose not to answer**	9	2.4	5	2.5	4	2.2
**Annual Household Income**						
**Less than $15,000**	5	1.3	2	1.0	3	1.7
**$15,000—$29,999**	8	2.1	4	2.0	4	2.2
**$30,000—$59,999**	43	11.4	23	11.5	20	11.2
**$60,000—$99,999**	118	31.2	62	31.0	56	31.5

**Table 8:** Derivation of RUFIT-NZ program costs

Cost parameter	Value
Coaching	$40,293
Printing	$3,327
Phone	$120
Travel	$1,365
T-shirts	$4,825
Advertising	$2,714
Staff costs	$26,109
**Total intervention cost**	**$77,469**
**Cost per participant**	**$752**

## Abbreviations

AUDIT: Alcohol use disorders identification test
AUDIT-C: Alcohol use disorders identification test-consumption
BCTs: Behavior change techniques
BMI: Boddy mass index
BVCF: Baseline value carried forward
CI: Confidence interval
CM: Centimeter
CONSORT: Consolidated standards of reporting trials
CPI: Consumer price index
FAB: Family, Activity, behavior
FFIT: Football Fans in Training
HRQoL: Health related quality of life
ICER: Incremental cost-effectiveness ratio
ITT: Intention to treat
Kg: Kilogram
MI: Multiple imputation
mmHg: Millimeters of mercury
NZ: New Zealand
OR: Odds ratio
PAR-Q: Physical activity readiness-questionnaire
PP: Per protocol
RCT: Randomized controlled trial
RPE: Rating of perceived exertion
RUFIT-NZ: Rugby Fans in Training-New Zealand
SAAFE: Supportive, active, autonomous, fair, enjoyable
SD: Standard deviation
